# A randomised open-label trial to assess outpatient induction of labour (HOMEIND) and compare efficacy of Propess vs Dilapan-S® for induction of labour at 39 weeks’ gestation in normal risk nulliparous women: study protocol for a randomised controlled trial

**DOI:** 10.1186/s13063-023-07174-7

**Published:** 2023-02-23

**Authors:** Sarah M. Nicholson, Orla Smith, Susan Hatt, Zara Molphy, Patrick Dicker, Karen Flood, Fergal Malone

**Affiliations:** 1 Royal College of Surgeons in Ireland, Dublin, Ireland; 2 Rotunda Hospital, Dublin, Ireland

**Keywords:** Induction of labour, Outpatient induction, Dilapan-S, Propess, Induction, Elective induction of labour, Cervical priming, Osmotic dilator, Cervical ripening

## Abstract

**Background:**

Induction of labour (IOL) at 39 weeks has been shown to decrease maternal and neonatal adverse outcomes. Given the growing demand for 39-week IOL, it is imperative that effective methods be assessed for induction in the outpatient setting.

The aim of this study is to answer the clinical question as to whether Dilapan-S® vs Propess® as a method of cervical ripening is non-inferior in the outpatient setting at 39 weeks and to ascertain whether Dilapan-S® 12 h is non-inferior to Dilapan-S® 24 h.

**Methods:**

This study is an open-label parallel group single-centre *randomised* trial.

Participants are normal risk nulliparous women who have no pregnancy-related or medical contraindication to IOL. Women will be randomised to one of three induction groups—Dilapan-S® (12-h insertion or 24-h insertion) or Propess. Induction will be initiated between 39+0 and 39+4 weeks’ gestation and participants will return home for either 12 or 24 h. They will be readmitted 12/24 h later in order to continue with induction of labour.

Patient recruitment will take place over 30 months within a single centre. The study will recruit a maximum 109 women for each study arm. Total duration of participants’ involvement in the trial will be 8 weeks to allow for postpartum follow-up.

**Discussion:**

This study will definitively answer whether Dilapan-S is non-inferior to Propess® as a method of induction of labour in the outpatient setting and whether cervical ripening with Dilapan-S over a 12-h timeframe is non-inferior to cervical ripening with Dilapan-S over a 24-h timeframe.

**Trial registration:**

EudraCT Number 2019-004697-25 Registered 14 September 2020

## Administrative information

Note: the numbers in curly brackets in this protocol refer to SPIRIT checklist item numbers. The order of the items has been modified to group similar items (see http://www.equator-network.org/reporting-guidelines/spirit-2013-statement-defining-standard-protocol-items-for-clinical-trials/).

**Table:** 

Title {1}	The Home Induction trial: A randomised open-label trial to assess outpatient induction of labour, and compare efficacy of Propess vs Dilapan-S® for induction of labour at 39 weeks’ gestation in normal risk nulliparous women.
Trial registration {2a and 2b}.	EudraCT Number 2019-004697-25 Registered 14/09/2020
Protocol version {3}	V5 dated 28-November-2022
Funding {4}	Funding was received from The Rotunda Foundation. An educational grant was provided to the research department by Medicem S.R.O. The trial was peer-reviewed by the funding body. The funder has no further role in the study.
Author details {5a}	Dr. Sarah M. Nicholson, Royal College of Surgeons in Ireland Rotunda Hospital, Dublin Susan Hatt Royal College of Surgeons in Ireland Dr. Zara Molphy Royal College of Surgeons in Ireland Dr. Orla Smith Rotunda Hospital, Dublin Dr. Patrick Dicker Royal College of Surgeons in Ireland Dr. Karen Flood Royal College of Surgeons in Ireland Rotunda Hospital, Dublin Prof. Fergal Malone Royal College of Surgeons in Ireland Rotunda Hospital, Dublin
Name and contact information for the trial sponsor {5b}	Royal College of Surgeons in Ireland 123 St Stephen’s Green, Dublin 2
Role of sponsor {5c}	The sponsor has no role in trial design. The sponsor will have no role in: data collection; data analysis; data interpretation; report writing; or the decision to submit the manuscript for publication.

## Introduction

### Background and rationale {6a}

Induction of labour (IOL) has become a topical subject in recent years, with studies examining the role of IOL at certain gestations in achieving lower caesarean section rates, reduced neonatal morbidity and mortality rates, and improved maternal experience. These outcomes have been examined through RCTs and observational and qualitative studies amongst others.

It has been shown that IOL at 39 weeks’ gestation in otherwise uncomplicated (normal risk) nulliparous women decreases the primary caesarean section rate, is cost-effective, does not result in increased perinatal adverse outcomes, and may decrease some adverse outcomes [1–3]. This research has shown that pain scores were reported as lower, and perceived control scores (i.e. expectations and experiences of personal control during childbirth as measured with the Labor Agentry Scale) were reported as higher (indicating greater perceived control) with IOL at 39 weeks compared to expectant management [1].

What is not clear from the literature is whether there is a more effective method of achieving successful IOL in such patients at 39 weeks, and if the cervical ripening process can be managed outside of the hospital setting, ideally reducing resource utilisation and resulting in a more positive experience within the cohort being induced.

A vaginal dinoprostone delivery system (Propess or Cervidil) is a common method of cervical ripening for induction of labour, in which a delayed-release prostaglandin formulation is placed within the vagina. Dilapan-S is a synthetic osmotic hygroscopic gel which acts as a mechanical induction agent which has also been utilised in the setting of IOL and works by absorbing moisture from surrounding tissues to gradually dilate the cervix by expansion. Whilst both have been the subject of a number of studies assessing efficacy and safety, no trial has compared the efficacy of both in relation to each other at 39 weeks in the outpatient setting. Given the impact of the ARRIVE Trial, which suggests increased utilisation of IOL, and given the capacity restrictions on inpatient labour ward settings, there is now an urgent requirement to evaluate the optimal method of outpatient cervical ripening in order to support this likely increased demand.

The IND HOME trial aims to assess the two methods of IOL, both of which allow the patient to return home for 12 or 24 h after initiation. The primary outcome of the study is assessment of vaginal delivery by any means within a given time frame (36 h in the 12-h Dilapan-S® group and 48 h in the 24-h Dilapan-S® and Propess® groups), assessing suitability of both methods as cervical ripening agents in an outpatient setting.

### Objectives {7}

#### Primary objective

To demonstrate non-inferiority in the efficacy of Dilapan-S® (12 h or 24 h insertion) to Propess for outpatient induction of labour at 39 weeks’ gestation in otherwise uncomplicated, normal risk* nulliparous women.

*A pregnancy is considered “normal-risk” when there are no active complications and there are no maternal or fetal factors that place the pregnancy at increased risk for complications. Specifically, the following conditions should be met to consider a pregnancy to be normal risk:

- Singleton pregnancy
- Cephalic presentation
- Term gestation (37–39 weeks gestational age)
- Maternal pre-pregnancy body mass index < 35 kg/m^2^
- Maternal age of ≥ 18 and < 40 years
- No evidence of the following conditions:
  - ◦ Pre-pregnancy diabetes
  - ◦ Gestational diabetes
  - ◦ Pre-pregnancy hypertension
  - ◦ Preeclampsia
  - ◦ IUGR
  - ◦ Oligohydramnios
  - ◦ Cervical cerclage in situ
  - ◦ Premature rupture of membranes
  - ◦ Congenital fetal anomalies
- Absence of other active obstetric complication not mentioned above

#### Secondary objective(s)

1. To compare the Dilapan-S® (12 h) group to the Propess group in the primary endpoint and secondary endpoints.
2. To compare the Dilapan-S® (24 h) group to the Propess group in the primary endpoint and secondary endpoints.
3. To compare the Dilapan-S® (12 h) group to the Dilapan-S® (24 h) group in the primary endpoint and secondary endpoints.
4. To compare rates of vaginal delivery in the Dilapan-S® 12-h group, the Dilapan-S® 24-h group, and the Propess group at 36 h, 48 h, and 48 h respectively from insertion time to delivery.

### Trial design {8}

This study is an open-label parallel group single-centre non-inferiority trial.

Participants are normal risk nulliparous women who have no pregnancy-related or medical contraindication to IOL. Women will be randomised to one of three induction groups—Dilapan-S® (12-h insertion or 24-h insertion) or Propess, which will be initiated between 39+0 and 39+4 weeks’ gestation, and then allowed to return home for either 12 or 24 h. They will be readmitted 12/24 h later and reassessed in order to continue with induction of labour.

Patient recruitment will take place over 30 months within a single centre. The study will recruit a maximum 109 women for each study arm. Total duration of participants’ involvement in the trial will be 8 weeks to allow for postpartum follow-up.

## Methods: participants, interventions, and outcomes

### Study setting {9}

This trial will be conducted at the Rotunda Hospital, a large tertiary referral centre in Dublin, Ireland.

### Eligibility criteria {10}

#### Inclusion criteria

In order to be eligible to participate in this study, an individual must meet all of the following criteria:

- Provide signed and dated informed consent form
- Willing to comply with all study procedures and be available for the duration of the study
- Normal risk nulliparous women (as defined in trial objectives)
- Age ≥ 18 and < 40 years
- Singleton pregnancy
- No contraindications to induction of labour
- Must agree to outpatient induction at 39 weeks
- No relevant medical issues in or outside of pregnancy
- Must live within 30 min or 15 km of hospital and have transport to hospital at all times during induction period
- Have a normal amniotic fluid index (AFI) at 39 weeks’ gestation (between 8 cm and 20 cm)
- Have a biophysical Profile Score (BPS) of 8/8
- Have a Bishop score < 6 at visit 2

#### Exclusion criteria

An individual who meets any of the following criteria will be excluded from participation in this study:

- Multiparous women
- Women with uterine scar
- Women with low lying placenta
- Women with BMI > 35 kg/m^2^
- Multiple gestation
- Known fetal anomaly or fetal growth restriction or oligohydramnios
- Known maternal health problem, e.g. hypertensive disease, cardiac disease, renal disease, diabetes, pulmonary disease, hepatic disease which would directly affect the risk status of the woman. This is assessed clinically on a case-by-case basis.
- Women with no transport to hospital or women who live > 30 min or > 15 km from the hospital
- Patients who have difficulty understanding the required protocol and follow*-*up instructions (e.g. language barriers)
- Women < 39+0 or greater than 39+4 weeks’ gestation at the time of induction of labour (visit 2)
  - ◦ Gestational age will be based on initial dating scan between 7 and 14 weeks, which confirms gestational age by CRL
- Any factor which is a contraindication to induction of labour
- Contraindications to trial treatment include patients that fall into any of the following categories:
  - ◦ If labour has started
  - ◦ If oxytocic drugs and/or other labour induction agents have *been* given
  - ◦ When strong prolonged uterine contractions would be inappropriate
  - ◦ Patients who have had previous major uterine surgery, e.g. caesarean section, myomectomy
  - ◦ Patients with a suspicion for cephalopelvic disproportion
  - Patients with fetal malpresentation
  - ◦ Patients with suspicion or evidence of non-reassuring fetal testing
  - ◦ Who have had previous major surgery (e.g. other than biopsies and cervical abrasion) or rupture of the uterine cervix
  - ◦ When there is current pelvic inflammatory disease, unless adequate prior treatment has been instituted
  - ◦ Patients with hypersensitivity to dinoprostone or to any of the excipients listed in the Summary of Product Characteristics (SmPC) for Propess/Prostin
  - ◦ Patients with placenta previa or unexplained vaginal bleeding during the current pregnancy
  - ◦ Patients with evidence of infection, including genital tract infection

### Who will take informed consent? {26a}

It is the responsibility of the principal investigator at the recruitment site or another investigator delegated by the principal investigator to obtain written informed consent from each subject prior to participation in the trial, following adequate explanation of the aims, methods, anticipated benefits, and potential hazards of the study. Adequate time must be given for consideration by the patient before taking part.

The investigator or designee will explain that patients are under no obligation to enter the trial and that they can withdraw at any time during the trial, without having to give a reason for withdrawal. No clinical trial procedures will be conducted prior to taking consent from the participant. Consent will not denote enrolment into the trial.

A copy of the signed informed consent form will be given to the participant. The original signed form will be retained in the investigator site file (ISF) at the study site and a copy placed in the medical notes. The participant information materials and informed consent form are available from the corresponding author on request.

If new safety information results in significant changes in the risk/benefit assessment, the patient information leaflet will be reviewed and updated if necessary and subjects will be provided with the new patient information leaflet (PIL) in a timely manner and new consent obtained.

### Additional consent provisions for collection and use of participant data and biological specimens {26b}

N/A—no additional data or biological specimens are taken for the purposes of this trial.

### Interventions

#### Explanation for the choice of comparators {6b}

A vaginal dinoprostone delivery system (Propess) is a common method of cervical ripening for induction of labour. Dilapan-S is a synthetic osmotic hygroscopic gel which acts as a mechanical induction agent which has also been utilised in the setting of IOL. Whilst both have been the subject of a number of studies assessing efficacy and safety, no trial has compared the efficacy of both in relation to each other at 39 weeks in the outpatient setting.

#### Intervention description {11a}

Eligible patients will be prescreened from antenatal clinic lists and contacted to gauge interest in participating. An information leaflet will be sent to any interested parties. If the patient is interested having read the information, a clinic date will be assigned at approximately 38 weeks.

Visit 1: Clinic. The patient will attend a specialised home induction antenatal clinic appointment at approximately 38 weeks’ gestation. They will have vital signs measured and urinalysis carried out. A growth scan and fetal wellbeing scan will be performed by a sub investigator, and eligibility criteria will be assessed. Informed consent will be obtained from patients who meet inclusion criteria and do not meet exclusion criteria. They will then be randomised to one of 3 groups: Dilapan-S 12 h, Dilapan-S 24 h, or Propess 24 h. They will be allocated a date for IOL from 39+0 to 39+4 weeks’ gestation. Patients will be informed of their allocated group in advance of their allocated induction date.

Visit 1: Recruitment. Baseline assessments include:

- Obtain medical history
- Vital signs
- Physical examination (if symptom driven)
- Abdominal examination
- Weight, height, BMI
- Urinalysis
- Ultrasound
- Randomisation

Visit 2: Induction. Patients will be admitted to the hospital on the assigned induction date and will have vital signs and urinalysis performed. They will undergo a scan to assess biophysical profile. All eligibility criteria will be reassessed before commencement of IOL. Cardiotocograph (CTG) monitoring will be performed for at least 20 min prior to commencing IOL. The cervix will be examined, and Bishop score will be determined. Provided patients are still eligible; IOL will be carried out with the allocated device as per manufacturer’s instructions. CTG monitoring will be performed for 30 min (Dilapan-S) or 60 min (Propess) following application of the relevant intervention. Participants will then go home with a patient safety checklist and instructions for monitoring at home and instructions on when to return to hospital. Two phone calls will be made to the patients whilst at home to assess patient wellbeing.

Visit 2 assessments will include:

- Vital signs
- Urinalysis
- Bishop score
- Ultrasound
- Physical examination (if symptom driven)
- Abdominal examination
- Investigational medicinal product (IMP) administration
- Adverse event review
- Concomitant medication review
- CTG

Visit 3: Reassessment. Patients will return to hospital at the allocated time, or before if clinically necessary (e.g. spontaneous rupture of membranes, vaginal bleeding, concerns re fetal movements or regular contractions). Vital signs will be measured, and urinalysis will be performed. A CTG will be performed. The IMP will be removed as per manufacturer’s guidelines, and reassessment of the cervix will be performed, and Bishop score will be determined. Clinical staff will assess whether the patient is suitable for artificial rupture of the membranes (ARM) or whether further ripening is needed. If further ripening is needed, Prostin gel at the dose of 1 mg will be given, a CTG will be performed, and the patient will be reassessed approximately 6 h thereafter. If deemed suitable for ARM, the patient will undergo same, a CTG will be performed, and the patient will be transferred to the labour ward when space is available. If required, oxytocin will be used to induce or augment labour as per hospital guidelines.

Visit 3 assessments will include:

- Vital signs
- Bishop score
- Physical examination (if symptom driven)
- Abdominal examination
- Adverse event review
- Concomitant medication review
- CTG

Visit 4: Delivery visit. Data is recorded from the electronic chart as per the schedule of events. Following delivery, a Maternal Satisfaction Questionnaire and stamped addressed envelope are sent to all participants to assess their experience throughout the trial.

Visit 4 assessments will include:

- Vital signs
- Physical examination (if symptom driven)
- Delivery details
- Maternal Satisfaction Questionnaire
- Neonatal outcomes
- Adverse event review
- Concomitant medication review

Visit 5: Six weeks following delivery, the participants are contacted, and data is recorded as per the schedule of events. This concludes the patient’s participation in the trial.

Visit 5 assessments will include:

- Maternal Satisfaction Questionnaire *(if unable to complete at visit 4)*
- Neonatal outcomes (if there has been a change since visit 4)
- Adverse event review
- Concomitant medication review

#### Criteria for discontinuing or modifying allocated interventions {11b}

Participants will be withdrawn from the trial if:

- The patient requests to be withdrawn from the trial
- The patient is deemed unsuitable for outpatient management.
- If it is deemed unsafe to allow the participant to return home once the IOL has commenced.
- Recording of any withdrawals will take place including the reason for withdrawal and the circumstances around the withdrawal.
- The trial will be stopped if there are any concerns for patient safety.

#### Strategies to improve adherence to interventions {11c}

NA—Compliance with medication is not applicable as administration of both IMPs are performed within a clinical setting by the treating investigator.

#### Relevant concomitant care permitted or prohibited during the trial {11d}

N/A—There are no additional needs or prohibitions for the purposes of this trial once patient fits inclusion criteria.

#### Provisions for post-trial care {30}

The Rotunda Hospital (sponsor) will indemnify claims from participants for injury caused by their participation in the clinical trial*.* However, as this clinical trial is being carried out at a hospital site, the hospital will continue to have a duty of care to the participants of the clinical trial.

There will be no arrangements to provide the IMPs to trial participants post trial as this study is specific to IOL.

#### Outcomes {12}

For all study participants, information will be recorded in the electronic chart as is standard for all patients in the hospital. Data on all of the above primary and secondary objectives will be collected and collated.

At the initiation of induction, the gestational age and exact time of initiation of induction will be recorded.

A questionnaire and stamped addressed envelope will be sent to participants to complete following delivery, in order to assess their satisfaction scores with the process of outpatient induction.

##### Primary outcome measure

The primary outcome measure (efficacy measure) is failure to achieve vaginal delivery (or, equivalently, operative vaginal delivery or SVD) at any time. This will allow assessment of effective methods of IOL in the outpatient setting. The window for induction will be 39+0 to 39+4 weeks gestation.

##### Secondary outcome measures

1. Overall change in Bishop score before and after cervical ripening
2. Rates of vaginal delivery at 36 h after insertion of either Propess or Dilapan-S®
3. Rates of vaginal delivery at 48 h after insertion of either Propess or Dilapan-S®
4. The need for second induction modality
5. Rates of hyper-stimulation*
6. Rate of failed induction
7. Overall length of stay in hospital
8. Rates of adverse neonatal outcome
9. Rates of adverse maternal outcomes
10. Maternal satisfaction scores with the outpatient induction process
11. Caesarean delivery rates, categorised by “overall rate”, “rate for failure to progress/failed induction”, and “rate for non-reassuring fetal testing”
12. Analgesia use in each group, including rates of epidural
13. Compare rates of 39 weeks’ successful vaginal delivery in the outpatient setting to rates of successful vaginal delivery in the inpatient setting**.

*Defined as more than 7 contractions in a 15-min time period persistently for more than 30 min and requiring a medical intervention (such as a clinical decision to remove the Propess/Dilapan or administer a medication such as Terbutaline)

**Defined as a group of low-risk nulliparous women who did not undertake elective induction of labour at 39 weeks as part of this trial

The primary objectives will therefore assess the efficacy of achieving labour with each of the study induction methods. The secondary objectives will assess other efficacy measures, safety measures and maternal satisfaction scores which are important factors in deciding whether this is a feasible option long term, and risk stratification in this process. Although many studies have shown both induction mediums to be low risk, it is important to compare the outcomes as above in order to guide the best possible IOL methods and create the most appropriate protocol for women to undergo IOL.

#### Participant timeline {13}

**Fig. 1:**
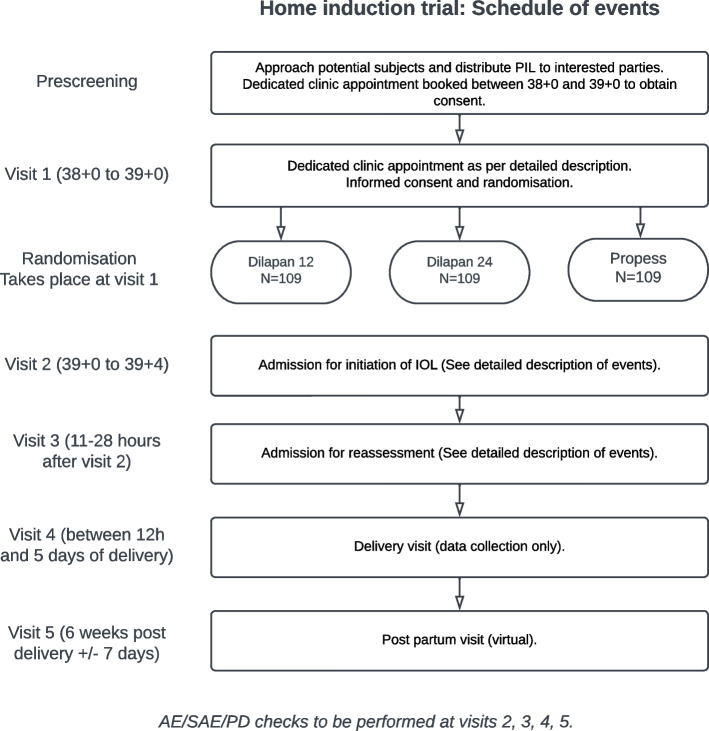
Schedule of assessments timeline for HOME IND trial

The participant timeline is shown in Fig. 1.

#### Sample size {14}

A single primary comparison will be used to determine efficacy. The sample size was therefore revised, from a potentially under-powered study of two co-primary comparisons, to an adequately powered study of a single primary comparison.

A 10% margin was used as the non-inferiority margin for the treatment comparison of Dilapan-S (combined groups) versus Propess. SVD rates were assumed to be 70%, 75%, and 80% in the Propess, Dilapan-S® (24 h), and Dilapan-S® (12 h) groups, respectively. Assuming 90% statistical power, a one-sided 2.5% level of statistical significance, and equal treatment group allocation, the study sample size required is 285 for a per-protocol analysis. Allowing for a 15% non-adherence rate, the total required recruitment is 327 (109 per group) for the intention-to-treat analysis. Other treatment comparisons will be considered secondary and exploratory.

#### Recruitment {15}

This trial will be conducted at one hospital site. Identification of potential subjects will be from the antenatal clinic lists. Potential participants who may wish to enquire about the study through advertising will also be able to contact the investigator.

There are approximately 3000 eligible patients per year attending the Rotunda as published annually in the hospital reports. Similar trials in the past have shown an uptake rate of 30% for inpatient induction of labour. With a more conservative uptake rate of about 20% (3000 × 20% = 600), we would therefore expect to recruit the required number of participants within 30 months (recruitment will have a maximum allowable duration of 30 months), allowing for dropout rates and those that fit exclusion criteria.

### Assignment of interventions: allocation

#### Sequence generation {16a}

Patients will be randomised using simple random allocation to either Dilapan-S® (12-h insertion or 24-h insertion) or Propess in a 1:1:1 ratio using a block size of 6 via a computer-generated randomisation procedure (software SAS Version 6.4).

#### Concealment mechanism {16b}

The use of a validated password website by the pharmacy department, not the investigator, will ensure concealment.

#### Implementation {16c}

A computer-generated randomisation sheet will be used to assign study participants to either Propess, Dilapan-S® 12 h, or Dilapan-S® 24 h groups at visit 1. The randomisation sheet will provide notification of the treatment package to be assigned to the participant and a log of each randomisation will be retained. The randomisation will be performed by the pharmacy department and relayed to the investigator. Subsequent to screening and consent procedures, the participants will be randomised to one of the three groups. The randomisation will use a block size of 4, and the random number seed-generator and generating program will be retained for reproducibility. The software SAS 6.4 will be used to generate the randomisation list.

### Assignment of interventions: blinding

#### Who will be blinded {17a}

N/A—This is an open-label trial; therefore, there will be no blinding of investigators, medical staff, or statisticians on this trial.

#### Procedure for unblinding if needed {17b}

N/A—This design is open label; therefore, unblinding will not occur.

### Data collection and management

#### Plans for assessment and collection of outcomes {18a}

Data will be entered prospectively at the time of the visits into the patient’s electronic chart (source data) as is standard protocol within the hospital. All data will be collected by a member of the team from the electronic chart or filled questionnaires and entered into the *electronic case report form (eCRF)*.

Source data are all information, original records of clinical findings, observations, or other activities in a clinical trial necessary for the reconstruction and evaluation of the trial.

For data where no prior record exists and which are recorded directly in the eCRF, the eCRF will be considered the source document, unless otherwise indicated by the investigator.

In addition to the above, date(s) of conducting informed consent, trial number, study arm, and the fact that the patient is participating in a clinical trial *will* be added to the patients’ medical record contemporaneously.

It will be the responsibility of the investigator to ensure the accuracy of all data entered in the CRFs. The delegation log will identify all those personnel with responsibilities for data collection and handling, including those who have access to the trial database.

#### Plans to promote participant retention and complete follow-up {18b}

N/A—The IMP is given by the investigator at time of induction; therefore, no deviation is possible.

#### Data management {19}

Data handling will be in accordance with Data Protection Legislation. The study coordinator will be the data controller and will ensure data security, privacy (anonymisation), and archival, in accordance with the Hospital IT department data handling policies. The study data will be stored in a centralised and secured area and will not be distributed to a third party. The study data will only be accessed by study personnel, as required, for data monitoring and data analysis.

The software STATA 16 ICE will be used for data entry using pre-specified fields. In addition, the SAS Version 9.4 software will be used for querying data (anomaly detection and rectification), for DSMB reporting and for statistical analysis at study completion. A detailed Statistical Analysis Plan (SAP) will be finalised prior to last-patient last-visit.

#### Confidentiality {27}

All data will be handled in accordance with the applicable Data Protection legislation.

The case report forms (CRFs) will not bear the subject’s name or other personal identifiable data. The subject date of birth and trial identification number will be used for identification.

#### Plans for collection, laboratory evaluation, and storage of biological specimens for genetic or molecular analysis in this trial/future use {33}

N/A—No such specimens are collected in this trial.

### Statistical methods

#### Statistical methods for primary and secondary outcomes {20a}

The analysis of the primary outcome will use a confidence interval (95% level of confidence) for simple differences in proportions. Non-inferiority will be determined on the lower confidence interval limit (equivalent to a one-sided 97.5% confidence interval). Conditional on non-inferiority being demonstrated, superiority will be determined. The analyses will be performed in the per-protocol population, supported by an intention-to-treat analysis.

Secondary outcomes will be compared using confidence intervals and hypothesis tests. However, these will be considered exploratory in nature. No adjustment for multiple testing will be performed.

#### Interim analyses {21b}

N/A—No interim analysis will be performed.

#### Methods for additional analyses (e.g. subgroup analyses) {20b}

Multiple (multivariate) logistic regression for the primary endpoints to adjust for potential prognostic variables will be performed. Such analysis will be deemed exploratory in nature and not considered the primary analysis.

#### Methods in analysis to handle protocol non-adherence and any statistical methods to handle missing data {20c}

Missing data mechanisms will be explored with respect MNAR (missing not at random), and potential mechanisms associated will assessment biases will be explored statistically.

#### Plans to give access to the full protocol, participant-level data, and statistical code {31c}

The datasets generated and/or analysed during the current study are not publicly available due to limits set during the ethical review process but are available from the corresponding author on reasonable request.

### Oversight and monitoring

#### Composition of the coordinating centre and trial steering committee {5d}

The principal investigator (PI) will be responsible for selecting the members of the trial management group (TMG), trial steering committee (TSC), and data safety monitoring board (DSMB). The CI will organise the structure, frequency, and agenda of meetings. A member of the study coordinator office (RCSI) will sit on the TMG to provide advice and maintain trial oversight. The terms of reference for the TMG, TSC, and DSMB will be developed by the investigator, and the study coordinator and will be reviewed by the study coordinator prior to final approval to ensure they meet the requirements.

#### Trial management group (TMG)

The trial management group will meet two-monthly and be responsible for the day-to-day management of the trial and include the PI, a study coordinator representative collaborators, statistician, trial manager, and research assistants. The TMG will monitor all aspects of the conduct and progress of the trial, ensure that the protocol is adhered to, and take appropriate action to safeguard participants and the quality of the trial itself.

The TMG will report to the TSC and will ensure that the study is conducted in compliance with the protocol, GCP and applicable regulations. The responsibilities will include (but are not limited to):

- Reporting to the trial steering committee
- Identification of trial sites
- Confirmation that all approvals are in place before release of the IMP and the start of the trial at site
- Provision of training about the trial at site
- Provision of trial materials to the site
- Establishment of a data management centre
- 24-h advisory support
- Provision of regular information about the progress of the study to collaborators
- Response to any questions (e.g. from collaborators) about the trial
- Data security and quality and observation of data protection laws
- Safety reporting
- Assurance that trial is conducted in accordance with the ICH GCP
- Statistical analysis
- Publication of trial results

#### Data safety monitoring board (DSMB)

The outcome objective of the DSMB is to provide impartial and objective assessment of clinical trial safety data. Independence of the DSMB promotes:

- Greater objectivity relative to overall clinical benefit risk assessment
- Increased credibility of the clinical trial data
- Enables modification to trial, where necessary, in response to new external DSMB recommendation without introducing bias

The independent DSMB members will review clinical trial data to evaluate safety and scientific validity of the clinical trial. Review of the full safety data from the clinical trial will enable impartial conclusion and recommendation which may be to do one of the following:

- Continue with the clinical trial as planned
- Continue with the clinical trial but amend the protocol prior to moving to next phase of clinical trial
- Stop the clinical trial

#### Trial steering committee (TSC)

The TSC will be comprised of the TMG members with the addition of one independent person and will meet at a frequency outlined in the terms of reference. The role of the TSC is to oversee and supervise the progress of the clinical trial and ensure the clinical trial is being conducted in accordance with GCP and applicable regulations. The TSC will agree to any protocol amendments and provide advice to the CI on all aspects of the clinical trial. The TSC will make major decisions regarding the continuation of the clinical trial or substantial amendments to the protocol based on the recommendations of the DSMB and ethics committee. The responsibilities of the TSC will be documented in the Terms of Reference.

#### Composition of the data monitoring committee, its role and reporting structure {21a}

The study coordinator, RCSI, will assign an independent monitor who will visit the investigator intermittently to validate compliance of the protocol to *good clinical practice (GCP)*, the maintenance of the study related records, and the extensiveness and accuracy of a proportion of CRF entries compared to source data. The investigator will co-operate with the monitor to ensure that any potential discrepancies are resolved.

Monitoring procedures include a site initiation visit designed to clarify all prerequisites before the trial commences at the site, interim site monitoring visits, and study close-out visits. The study will be monitored by regular scheduled visits to site and on-going communication via telephone and e-mail.

During site visits, the monitor will review the original patient records for the patient group, CRFs, drug accountability records, investigator site file, and document retention. Study procedures will be observed by the monitor, and any issues will be discussed with the PI or designee as necessary.

At a minimum, source documentation will be available to substantiate subject identification, eligibility, and participation; proper informed consent procedures; dates of visits; adherence to protocol procedures; records of safety and efficacy parameters; adequate reporting and follow-up of AEs; administration of concomitant medication; drug receipt/dispensing/return records; study drug administration information; and dates of subject completion, discontinuation from treatment, or withdrawal from the study, including the reason if appropriate.

CRF entries will be verified with the source documentation, if applicable (in some cases there are no source pages, therefore verification is not necessary). If any data, signatures, or forms are missing or incorrect, the investigator or designee will be informed and corrections will be made. Direct access to all source documents must be guaranteed by the PI, who must provide support at all times for these activities.

#### Adverse event reporting and harms {22}

All adverse events (AEs) occurring during the study observed by the investigator or reported by the subject will be recorded on the eCRF, except for those events that meet the definition of a non-reportable event. All AEs will be recorded from visit 2 until the 6 weeks postpartum follow-up visit.

The following information will be recorded: description, date of onset and end date, severity, assessment of relatedness to the study medication, other suspect medication or device, and action taken and outcome. Follow-up information should be provided as necessary. All AEs will be recorded with clinical symptoms and accompanied with a simple, brief description of the event, including dates as appropriate.

If the investigator suspects that the subjects’ disease has progressed faster due to the administration of the IMP, then they will record and report this as an unexpected adverse event. It will be left to the investigator’s clinical judgement whether or not an AE is of sufficient severity to require the subject’s removal from treatment. A subject may also voluntarily withdraw from treatment due to what she perceives as an intolerable AE. If either of these occurs, the subject must undergo an end-of-study assessment and be given appropriate care under medical supervision until symptoms cease or the condition becomes stable.

All serious adverse events (SAEs) will be reported to RCSI pharmacovigilance, except for those events that meet the definition of a non-reportable event. The principal investigator (PI) or appropriate designee is responsible for capturing all SAEs on appropriate trial specific forms and reporting to RCSI Pharmacovigilance within 24 h of first becoming aware of the event*.*

SAEs will be collected from visit 2 until the 6 weeks postpartum consultation and will be followed until resolution or until stabilised with sequelae.

Regarding AE/SAE in the baby, all NICU admissions will be captured as adverse events, with investigators assessing each admission to verify if it meets criteria for a SAE. All AEs that meet SAE criteria will also be recorded as such as per protocol.

The study coordinator, RCSI will notify the main REC and competent authority of all suspected unexpected serious adverse reactions (SUSARs). SUSARs that are fatal or life-threatening must be notified to the CA and REC within 7 calendar days after the study coordinator has learned of them. Other SUSARs must be reported to the REC and CA within 15 calendar days after the study coordinator has learned of them.

#### Frequency and plans for auditing trial conduct {23}

The study coordinator, RCSI, will assign an independent monitor. Monitoring will be carried out throughout the trial period. Monitoring within this trial involves 100% consent form review for all subjects. In addition, a 33% in-depth review (source data verification) for all visits for 33% of 327 are assessed by the monitor.

In addition, as per HPRA guidelines, this trial was classified as an interventional clinical trial within the definition outlined in the legislation (SI No 190 of 2004) and therefore is subject to HPRA monitoring. The HPRA receive annual reports including all AE/SAEs that have taken place on the trial.

#### Plans for communicating important protocol amendments to relevant parties (e.g. trial participants, ethical committees) {25}

The HOME IND trial has been approved by the HPRA. Any protocol amendments will be submitted to the HPRA for approval and thereafter to the national ethics committee in the National Maternity Hospital before being instituted. All approved changes are relayed to all trial investigators and staff on approval, and confirmation from each member that updates have been noted is recorded by the trial coordinator.

#### Dissemination plans {31a}

The results will be submitted for publication in a peer reviewed journal after completion of the trial. In addition, the results may be presented at national and international meetings.

## Discussion

When the proposal and protocol were in development throughout 2019 and 2020, the profound effect of COVID-19 on our healthcare system was not anticipated. The impact of the pandemic resulted in significant logistical difficulties for the HOME IND trial. Recruitment policies relied on telephone calls rather than in-person meetings. Restricted visiting hours were an inhibitory factor for people who may have otherwise consented to enrolment. Development of an active COVID-19 infection led to multiple participants being excluded at visit 2, resulting in a number of participants who had been randomised never receiving the IMP. Overall, we believe that the COVID-19 pandemic had a detrimental effect on both recruitment and the running of the trial, leading to an extended recruitment time in comparison to our pre-pandemic predictions.

## Trial status

Current protocol version 5; 28 November 2022

Recruitment commenced 23 November 2020

Recruitment end (approximately) 15 June 2023

## Data Availability

The datasets generated and/or analysed during the current study are not publicly available due to limits set during the ethical review process but are available from the corresponding author on reasonable request.

## Abbreviations

AE: Adverse events
DSMB: Data safety monitoring board
eCRF: Electronic case report form
EudraCT: European Clinical Trials Database
HPRA: Health Product Regulatory Authority
HSE: Health Services Executive
IOL: Induction of labour
IMP: Investigational medicinal product
MNAR: *Missing not at random*
NICE: National Institute for Health and Care Excellence
NICU: Neonatal intensive care unit
PI: Principal investigator
PPH: Post-partum haemorrhage
RCT: Randomised controlled trial
SAE: Serious adverse event
SSA: Site-specific assessment
